# Adiponectin alleviated Alzheimer‐like pathologies via autophagy‐lysosomal activation

**DOI:** 10.1111/acel.13514

**Published:** 2021-11-14

**Authors:** Kaiwu He, Lulin Nie, Tahir Ali, Shujin Wang, Xiao Chen, Zizhen Liu, Weifen Li, Kaiqin Zhang, Jia Xu, Jianjun Liu, Zhi‐Jian Yu, Xifei Yang, Shupeng Li

**Affiliations:** ^1^ State Key Laboratory of Oncogenomics School of Chemical Biology and Biotechnology Peking University Shenzhen Graduate School Shenzhen China; ^2^ Shenzhen Key Laboratory of Modern Toxicology Shenzhen Medical Key Discipline of Health Toxicology Shenzhen Center for Disease Control and Prevention Shenzhen China; ^3^ Department of Neurology the First People’s Hospital of Zibo Affiliated to Weifang Medical College Zibo China; ^4^ College of Public Health University of South China Hengyang China; ^5^ Department of Pathophysiology Guangzhou Medical University Guangzhou China; ^6^ Department of Infectious Diseases and Shenzhen key laboratory for endogenous infections the 6th Affiliated Hospital of Shenzhen University Health Science Center Nanshan District Shenzhen China; ^7^ Campbell Research Institute Centre for Addiction and Mental Health Toronto Ontario Canada; ^8^ Department of Psychiatry University of Toronto Toronto Ontario Canada

**Keywords:** adiponectin, autophagy‐lysosomal pathway, cognitive impairments, dementia, neuroinflammation

## Abstract

Adiponectin (APN) deficiency has also been associated with Alzheimer‐like pathologies. Recent studies have illuminated the importance of APN signaling in reducing Aβ accumulation, and the Aβ elimination mechanism remains rudimentary. Therefore, we aimed to elucidate the APN role in reducing Aβ accumulation and its associated abnormalities by targeting autophagy and lysosomal protein changes. To assess, we performed a combined pharmacological and genetic approach while using preclinical models and human samples. Our results demonstrated that the APN level significantly diminished in the plasma of patients with dementia and 5xFAD mice (6 months old), which positively correlated with Mini‐Mental State Examination (MMSE), and negatively correlated with Clinical Dementia Rating (CDR), respectively. APN deficiency accelerated cognitive impairment, Aβ deposition, and neuroinflammation in 5xFAD mice (5xFAD*APN KO), which was significantly rescued by AdipoRon (AR) treatment. Furthermore, AR treatment also markedly reduced Aβ deposition and attenuated neuroinflammation in APP/PS1 mice without altering APP expression and processing. Interestingly, AR treatment triggered autophagy by mediating AMPK‐mTOR pathway signaling. Most importantly, APN deficiency dysregulated lysosomal enzymes level, which was recovered by AR administration. We further validated these changes by proteomic analysis. These findings reveal that APN is the negative regulator of Aβ deposition and its associated pathophysiologies. To eliminate Aβ both extra‐ and intracellular deposition, APN contributes via the autophagic/lysosomal pathway. It presents a therapeutic avenue for AD therapy by targeting autophagic and lysosomal signaling.

## Graphical abstract

Our study revealed that adiponectin (APN) deficiency accelerates Aβ deposition, neuroinflammation, and cognitive behaviors impairments. However, AdipoRon treatment could reverse these pathological changes. Further molecular studies show APN contributes to AD‐like deficits via the autophagy‐lysosomal pathway. Thus, APN is the negative regulator of Aβ deposition and its associated pathologies.

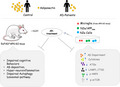

## 1 INTRODUCTION

Alzheimer's disease (AD) is the most common form of dementia affecting millions of people word widely, predicting to be tripled by 2050 according to the epidemiology studies ("Global, regional, and national burden of Alzheimer's disease and other dementias, 1990–2016: a systematic analysis for the Global Burden of Disease Study 2016," 2019; Kalaria et al., 2008; Nichols et al., 2019). Extracellular accumulation of β‐amyloid (Aβ) into amyloid plaques, intraneuronal hyperphosphorylated tau aggregation, neuroinflammation, synaptic loss, and neuronal cell death are standard features of AD. Further, a causative relationship between Aβ accumulation and AD pathology suggests that Aβ aggregation proceeds neurofibrillary tangles (NFTs) formation. However, NFT formation is closely related to the cognitive dysfunction of AD (Di et al., 2016; Nelson et al., 2009, 2012; Sadigh‐Eteghad et al., 2015; Zhang et al., 2018). Although the etiological roles of Aβ and NFTs have been challenged following the repetitive clinical trial failures of therapeutics targeting them. The detailed and comprehensive pathophysiological mechanisms under the progressive loss of neurons and synapses in AD are not adequately defined.

Apart from marked AD pathologies, increasing studies have been seeking early etiological changes and risk factors in AD, which contains at the high ranks of insulin resistance, dysregulated glucose/lipid metabolism, and oxidative stress (Chen et al., 2014; Willette et al., 2015). Aβ accumulation could impair insulin signaling while insulin modulates Aβ trafficking, release, and Aβ‐mediated synaptic loss, supporting a strong association between insulin signaling and Aβ metabolism (Avrahami et al., 2013; De Felice, 2013). Insulin resistance is prominent in AD patients as demonstrated by higher fasting plasma insulin, while reduced insulin‐provoked Aβ elevation is reported in AD (Neth & Craft, 2017).

Adiponectin (APN) is one of the most abundant circulatory adipocytokine (Esmaili et al., 2020; Yamauchi & Kadowaki, 2008). Initially identified as a regulator of glucose and lipid metabolism in the periphery, APN has insulin‐sensitizing (Ahlstrom et al., 2017), antioxidative (Detopoulou et al., 2010), and anti‐inflammatory (Ouchi & Walsh, 2007) effects under different pathological conditions. APN ameliorates insulin sensitivity via AMP‐activated protein kinase (AMPK) phosphorylation (Cheng et al., 2014). It can also activate insulin receptor substrate (IRS) via phosphorylation at its tyrosine residue, which promotes insulin signaling activities, including GSK3β and glucose uptake inhibition (Anderson et al., 2014; Cheng et al., 2014).

The role of APN in the central nervous system (CNS) is not well and comprehensively studied. However, adiponectin receptors (AdipoR1 and AdipoR2) express in the hippocampus, cortex, and hypothalamus of the brain (Rastegar et al., 2019; Thundyil et al., 2012). Lower levels of APN have been observed in cerebrospinal fluid (CSF) and brain tissues of AD patients (Ng et al., 2020), suggesting a possible involvement of APN in brain function. Further, APN‐deficient mice exhibited cognitive impairment (Rizzo et al., 2020) and depressive‐like behaviors (Liu et al., 2012), proposing the role of APN in the improvement of cognitive functions. A most recent study crossbred 5xFAD mice with APN KO mice to produce 5xFAD*APN KO mice and found increased Aβ deposition, neuroinflammation, and cognitive impairment, which could be reversed by AdipoRon (AR) treatment (Ng et al., 2020). However, the underlying mechanisms of APN deficiency accelerating AD‐like pathologies, how AR rescues these, and their relations with insulin signaling are yet to be investigated. Therefore, in our present investigation, we highlighted the mechanistic relationship between APN and AD‐like changes.

Our study demonstrated that APN deficiency accelerates AD‐like pathologies; however, AR (AdipoRon) treatment abates the phenomenon by decreasing Aβ deposition in APN‐deficient 5xFAD mice. Furthermore, AR treatment reduces cognitive impairments and the dysregulation in autophagy‐lysosomal pathways (ALP) protein expression, proposing that AR can be a potential therapeutic drug to treat AD‐associated pathologies.

## 2 RESULTS

### 2.1 Dementia correlates with APN deficiency

**FIGURE 1:**
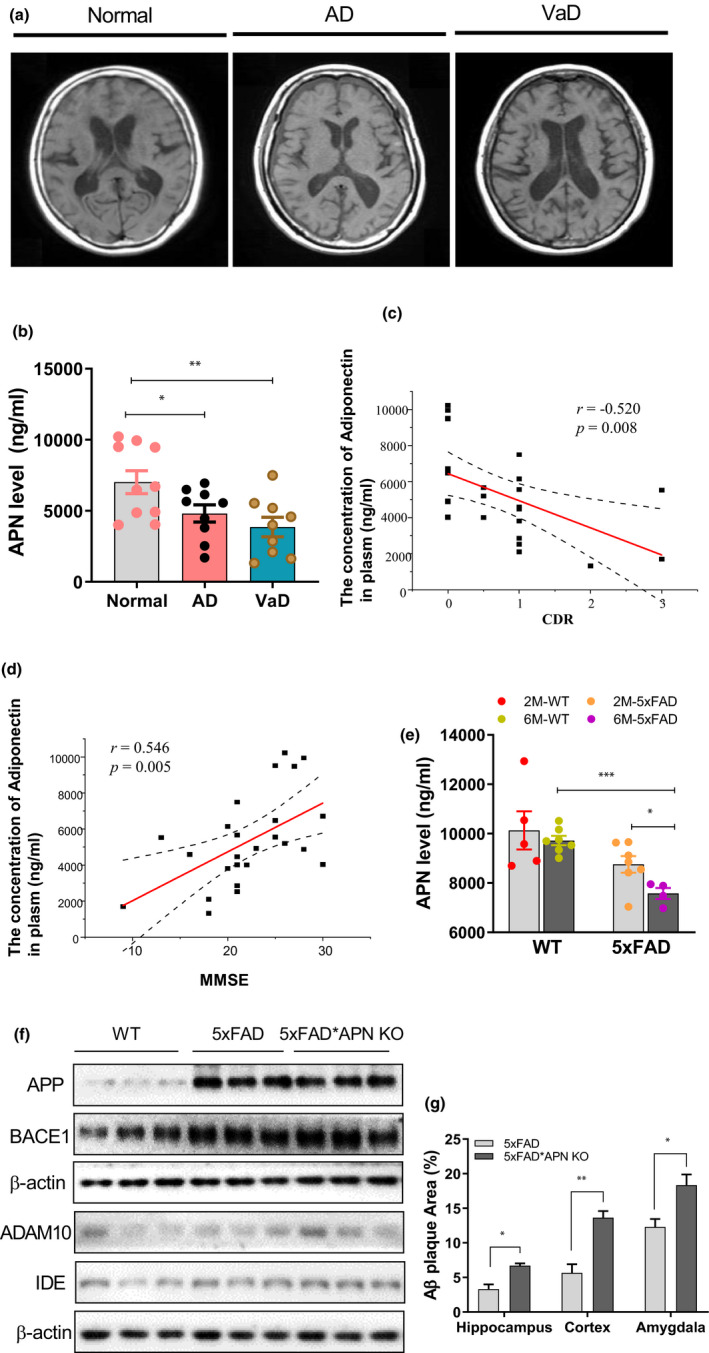
Dementia correlates with APN deficiency. (a) Original axial T1‐weighted MRI revealed medial temporal atrophy, including the hippocampus and frontotemporal cortex. (b) The level of APN in plasma of dementia patients and cognitively normal controls. (c) Correlation between APN concentration and CDR in each diagnostic group. (d) Correlation between APN concentration and MMSE in each diagnostic group. (e) The level of APN in plasma of 2/6‐month‐old 5xFAD mice and the controls. (f) The relative expression of APP, BACE1, ADAM10, and IDE in the hippocampus region. (g) Quantification of Aβ plaque in the hippocampus, cortex, and amygdala region of 5xFAD and 5xFAD*APN KO mice. Data were expressed as mean ± SEM, ^*^ *p* < 0.05, ^**^ *p* < 0.01, ^***^ *p* < 0.001

Age‐ and sex‐matched healthy individuals and dementia patients (AD and VaD: vascular dementia) were first selected as significant brain atrophy shown with MRI results (Table S1, S2; Figure 1a). Next, we examined plasma APN level, which was significantly reduced in dementia patients compared to healthy controls (Figure 1b; Figure S6A; Table S1, S2). Further, a significant linear correlation was detected between plasma APN concentration and CDR or MMSE score (Figure 1c, d), proposing a critical relationship between APN and AD. Interestingly, these correlations were also present in mice, as demonstrated by a significant decline of plasma APN level of 5xFAD mice compared to that of 2/6‐month‐old WT mice (Figure 1e). Notably, it is tentative to show that APN level reduction was accelerated with disease progression, as remarked APN level reduction could be examined in the plasma of 6‐month‐old 5xFAD mice compared to the 2‐month‐old 5xFAD mice (Figure 1e). These results collectively demonstrated a negative correlation of APN level and dementia progression, and these changes were conserved between mice and human pathological status.

### 2.2 APN deficiency accelerates Aβ deposition, accompanied by cognitive impairment and neuroinflammation

Previous studies have revealed that APN deficiency accelerates Aβ deposition (Ng et al., 2020; Rizzo et al., 2020); however, the mechanism is largely unknown. Here, we eliminate APN from 5xFAD mice, as shown in Figure S1A. No significant difference in APP expression was detected between 5xFAD*APN KO and 5xFAD mice (Figure 1f; Figure S1C). Similarly, no substantial changes in APP processing enzymes were revealed, including BACE1, ADAM10, and IDE (Figure 1f; Figure S1D‐F). Aβ deposition was validated with immunostaining. Results revealed that Aβ plaques accumulation accelerated in the cortex, hippocampus, and amygdala of 5xFAD*APN KO mice compared with 5xFAD mice (Figure 1g; Figure S1B). Together, these results demonstrated that APN deficiency accelerates Aβ deposition without affecting APP expression and processing in 5xFAD mice.

Next, we sought to determine the effect of APN deficiency on cognitive impairment in AD. 6‐month‐old mice were employed when impaired spatial memory has been reported in 5xFAD mice (Xiao et al., 2015). The mice were successively subjected to novel object recognition, Y‐maze, and morris water maze (Figure S2). The results showed APN‐deficient 5xFAD mice significantly reduced the new object preference compared to the WT mice (Figure S2A‐B). Moreover, APN deficiency aggravated spatial memory deficits of 5xFAD mice in the Y‐maze trial, as shown by a reduced percentage of time in the novel arm (Figure S2C). Similarly, in the Morris water maze test, APN deficiency accelerated learning and memory deficits in 5xFAD mice, as demonstrated by the increased latency to find the submerged platform during 5 consecutive days of training and the probe trial (Figure S2D‐I).

### 2.3 AR treatment reduced Aβ pathology and cognitive impairment

**FIGURE 2:**
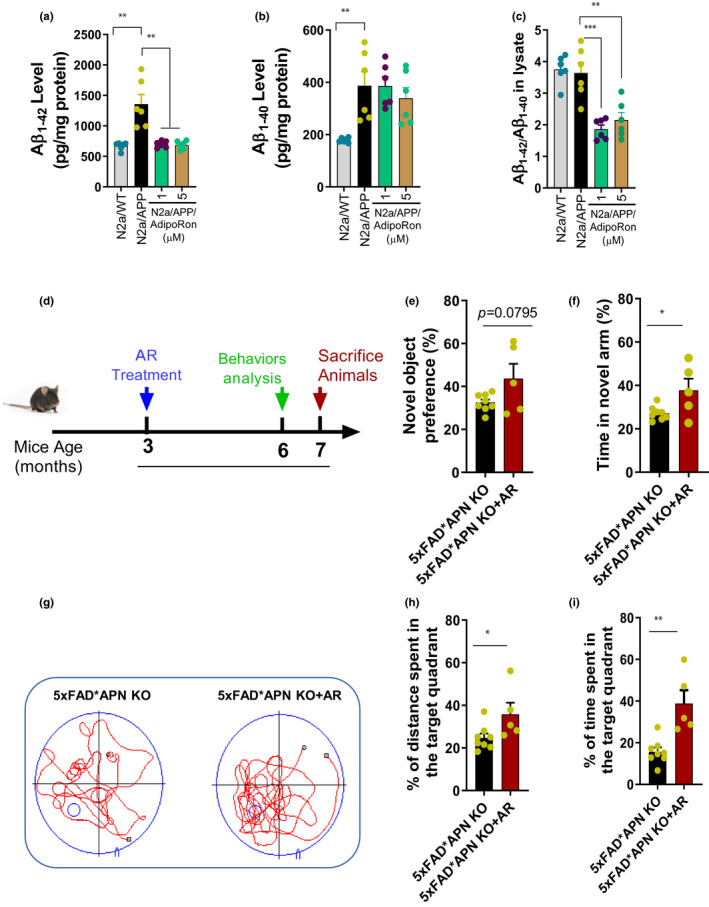
AR treatment reduced Aβ deposition and cognitive impairment. (a) The level of Aβ_1‐42_ in the cell lysate. (b) The level of Aβ_1‐40_ in the cell lysate. (c) The ratio of Aβ_1‐42_/Aβ_1‐40_. (d) 3‐month‐old 5xFAD*APN KO mice were orally treated with AR for 4 months. (e) The preference of the new object in the new object recognition test. (f) The percentage of time spent in a novel arm in the Y‐maze test. (g) The representative swimming trace in probe trial of morris water maze test. (h) The percentage of distance traveled in the target quadrant. (i) The percentage of time spent in the target quadrant. Data were expressed as mean ± SEM, ^*^ *p* < 0.05, ^**^ *p* < 0.01, ^***^ *p* < 0.001, ^****^ *p* < 0.0001

The possibility of the APN role in Aβ deposition was further validated by in vitro analysis with AdipoRon (AR, an AdipoR agonist) treatment. The results showed that "toxic" Aβ_1‐42_ protein was significantly higher in N2a/APP_swe_ lysates than N2a/WT, which were considerably reduced by 1µM or 5µM AR treatment (Figure 2a). However, we did not find, Aβ_1‐42_ expression in the supernatant of the N2a cells. Surprisingly, 5µM AR treatment markedly increased the Aβ_1‐40_ level in the lysates (but not in the supernatant: Figure S6B) of N2a/APP_swe_ (Figure 2b); however, the ratio of Aβ_1‐42_/Aβ_1‐40_ had significantly reduced by AR treatment in N2a/APP_swe_ cells (Figure 2c), further supporting the causal effect of APN in Aβ accumulation. Besides, we measured the AR effect on the Aβ plaques accumulation in the cortex and hippocampus of the 6‐month‐old APP/PS1 mice while treating with AR for two months. Interestingly, AR treatment significantly reduced Aβ plaques deposition in APP/PS1 mice (Figure S3A). The notion was further validated by immunostaining of the Aβ via 6E10 (Figure S6C). However, we did not detect any significant changes in the expression of crucial proteins (Figure S3B) involved in producing Aβ plaques.

As mentioned in the earlier results, APN deficiency increases cognitive impairment. Here, we also measured the experimental mice's cognitive skills after AR treatment (Figure 2d). By NOR and Y‐maze tests, we observed that AR treatment could attenuate the short‐term memory deficits of 5xFAD*APN KO mice (Figure 2e‐f). Further, the MWM test results also showed that the percentage of distance traveled and time spent in the target quadrant was also obviously increased after AR treatment (Figure 2g‐i). These findings suggested that AR treatment could rescue spatial memory deficits in 5xFAD*APN KO mice.

### 2.4 APN negatively regulates Aβ deposition via autophagy activation

**FIGURE 3:**
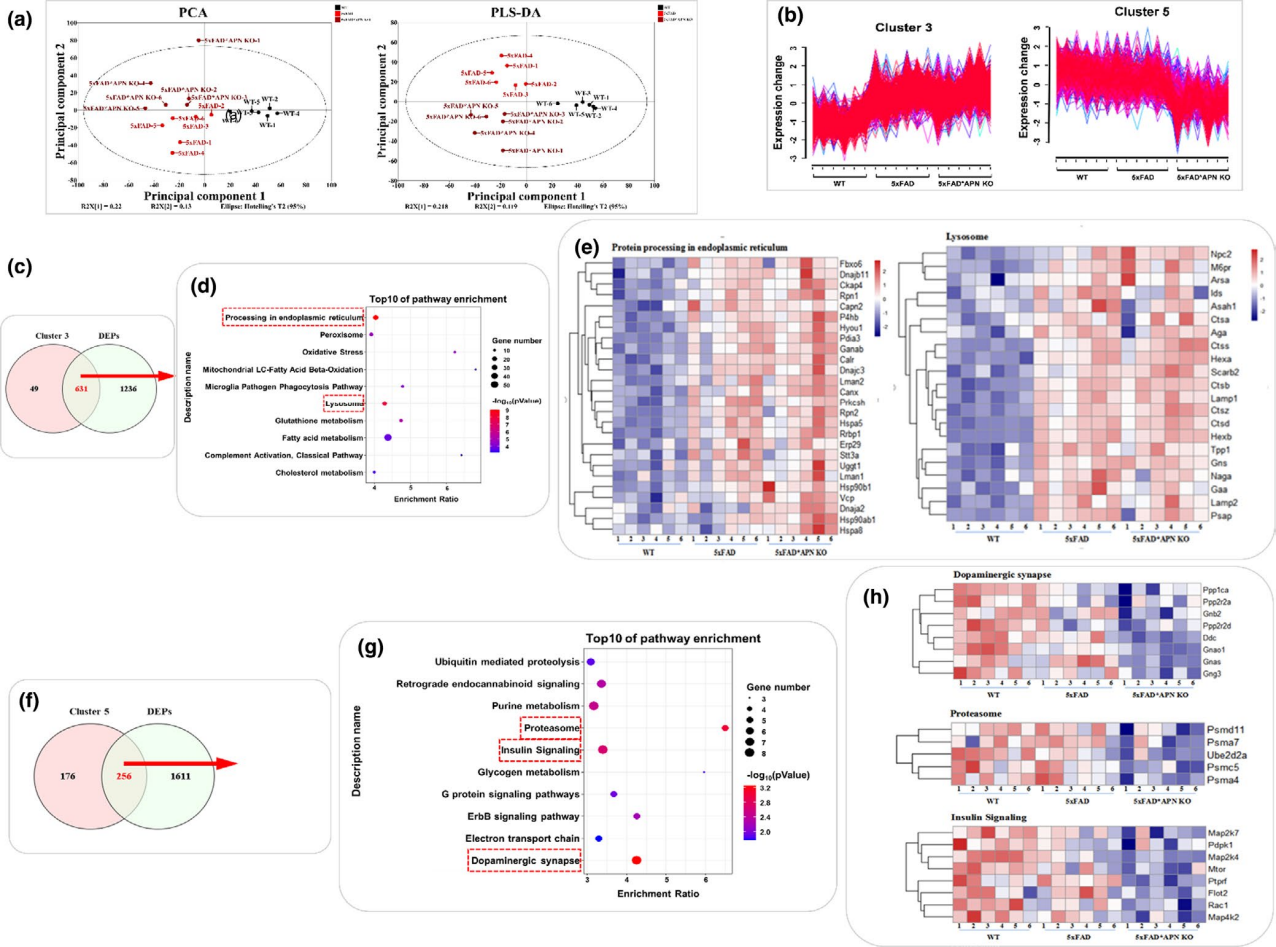
Proteome profiling overview of brain hippocampus in WT, 5xFAD, and 5xFAD*APN KO mice. Principal component analysis (PCA) and partial least squares‐discriminate analysis (PLS‐DA) of total proteins identified. (b) The expression patterns of cluster 3 and cluster 5. (c, f) The Venn diagram analysis between DEPs and cluster 3/5. (d, g) KEGG pathway analysis of 631 DEPs in cluster 3 and 256 DEPs in cluster 5. (e, h) The DEPs cluster 3 and 5 involved in the essential pathways were visualized by hierarchical heatmap clustering

To investigate the possible effects and underlying mechanisms of APN deficiency in AD, a proteomic analysis based on TMT‐labeled was performed, followed by PCA and PLS‐DA analysis (Figure 3a) (detail are in Supplementary data). A total of 5392 proteins were identified across the WT, 5xFAD, and 5xFAD*APN KO three groups. Among them, 2439 are the key proteins, and 1867 differentially expressed proteins (DEPs), including 1394 DEPs in 5xFAD vs WT group and 686 DEPs in 5xFAD*APN KO vs 5xFAD group. After being divided into 5 clusters, 3/5‐related potential vital proteins were identified, which might be the molecular basis for APN deficiency exacerbating AD cognitive impairment and pathology in 5xFAD mice (Figure 3b‐h; Figure S4). These results were further validated by KEGG enrichment analysis and hierarchical heatmap clustering analysis. Overall, higher connectivity was observed among neuron differentiation processes, neurogenesis, proteolysis, cell death, autophagy, and endocytosis in the brain tissue of 5xFAD*APN KO compared to 5xFAD WT mice, suggesting the above‐related process may play a crucial role underlying APN deficiency accelerated cognitive impairment.

**FIGURE 4:**
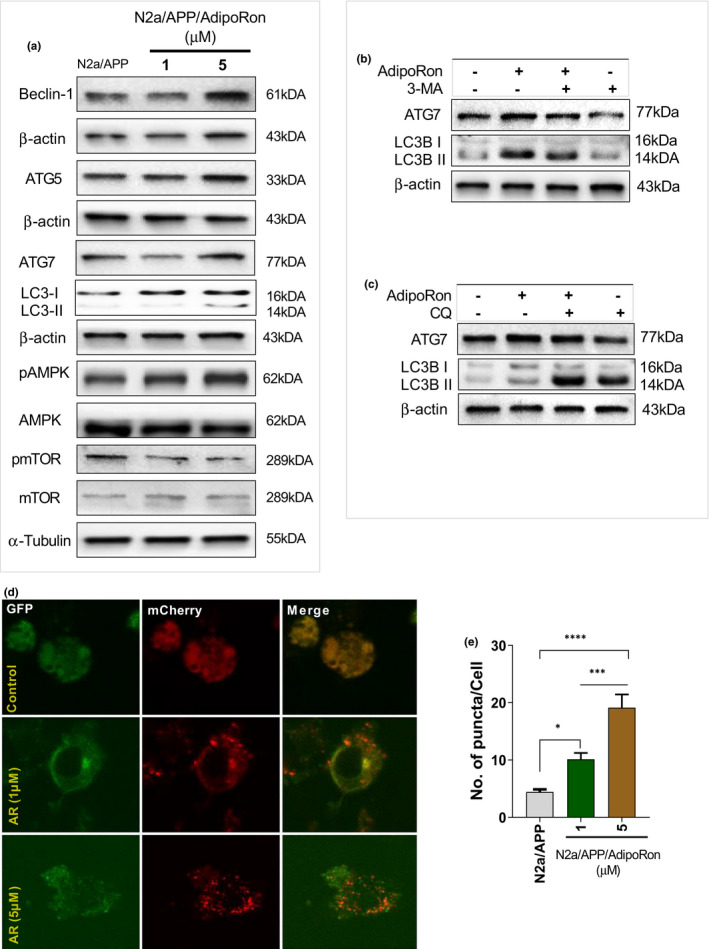
AR significantly triggered autophagy by mediating AMPK‐mTOR pathway signaling. The level of Beclin‐1, ATG5, ATG7, LC3II/LC3I, pAMPK/AMPK, and pmTOR/mTOR in N_2_aAPP_swe_ cells with or without AR treatment. (b) Autophagy inhibitor 3‐MA was used to suppress autophagy, representative Western blot analyses of LC3II and ATG7 levels. (c) Representative Western blot analyses of LC3II and ATG7 levels in the presence of CQ. (d‐e) Autophagosome images obtained with laser confocal microscopy after the N_2_aAPP_swe_ cells were transfected with a mCherry‐GFP‐LC3B plasmid and treated with AR for 48 hours. Data were expressed as mean ± SEM, ^*^ *p* < 0.05, ^***^ *p* < 0.001, ^****^ *p* < 0.0001

Former reports revealed that autophagy is essential to eliminate Aβ toxic accumulated (Li et al., 2017; Uddin et al., 2018). Here to associate, autophagy‐related gene (ATGs) expression was measured. As expected, ATGs, including Beclin‐1, ATG5, ATG7, and LC3‐II, were markedly increased in N2a/APP_swe_ cells treated with 5µM AR (Figure 4a; Figure S5A‐D), demonstrating autophagic activation of AR. Additionally, AR (5µM) treatment activated the AMPK‐mTOR signaling pathway by increasing p‐AMPK while decreasing mTOR phosphorylation in N2a/APP_swe_ cells (Figure 4a; Figure S5E‐F). These results were then corroborated with autophagy inhibitors, including 3‐MA and CQ, which selectively block AR's effects (Figure 4b‐c; Figure S5G‐J). Interestingly, autophagy activation by AR was further confirmed with enhanced LC3‐II changes, as demonstrated by increased the number of puncta in mCherry‐LC3 transfected N2a/APP_swe_ cells (Figure 4d‐e). These results indicated that AR significantly activated autophagy.

### 2.5 AR promoted Aβ clearance by increasing lysosome activity in microglia

**FIGURE 5:**
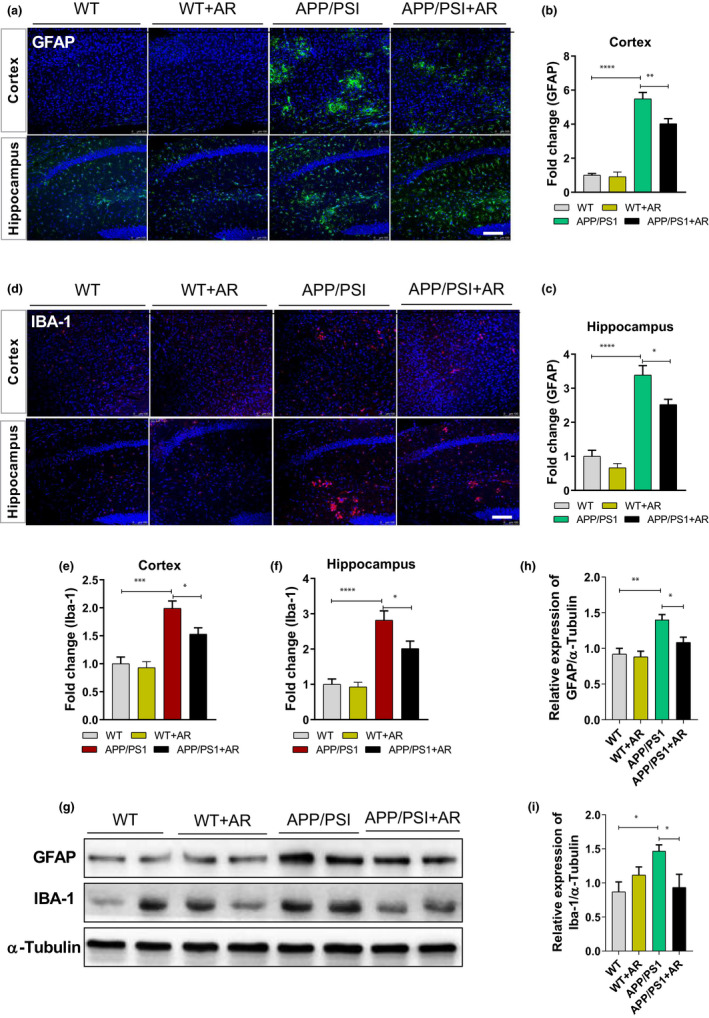
AR significantly reduced neuroinflammation in APP/PS1 mice. (a) Representative images of GFAP in the hippocampus and cortex of four group mice. (b‐c) Quantification of GFAP‐positive cells. (d) Representative images of Iba1 in the hippocampus and cortex of four group mice. (e‐f) Quantification of GFAP‐positive cells. (g, h) The relative expression of GFAP. (g, i) The relative expression of Iba1. Data were expressed as mean ± SEM, ^*^ *p* < 0.05, ^**^ *p* < 0.01, ^***^ *p* < 0.001, ^****^ *p* < 0.0001. Scale bar = 100 μm

Aβ (extracellular) deposition is mainly cleared through phagocytosis and microglia degradation, indicating that microglial activity is closely related to Aβ metabolism. Here, we measured GFAP and IBA‐1 levels, the glial cells activation markers, in the cortex and hippocampus. Significant increases were observed in both GFAP and IBA‐1 levels in the brain of the 5xFAD mice, which were remarkably elevated on APN deficiency (Figure S3C‐D). Additionally, AR treatment significantly reversed the hyper‐neuroinflammatory state of the APP/PS1 mice (Figure 5). These findings indicate that APN deficiency aggravated spatial learning and memory deficits in 5xFAD mice. Besides, AR treatment significantly reduced pro‐inflammatory cytokine levels in the N2a cells (Figure S7F).

**FIGURE 6:**
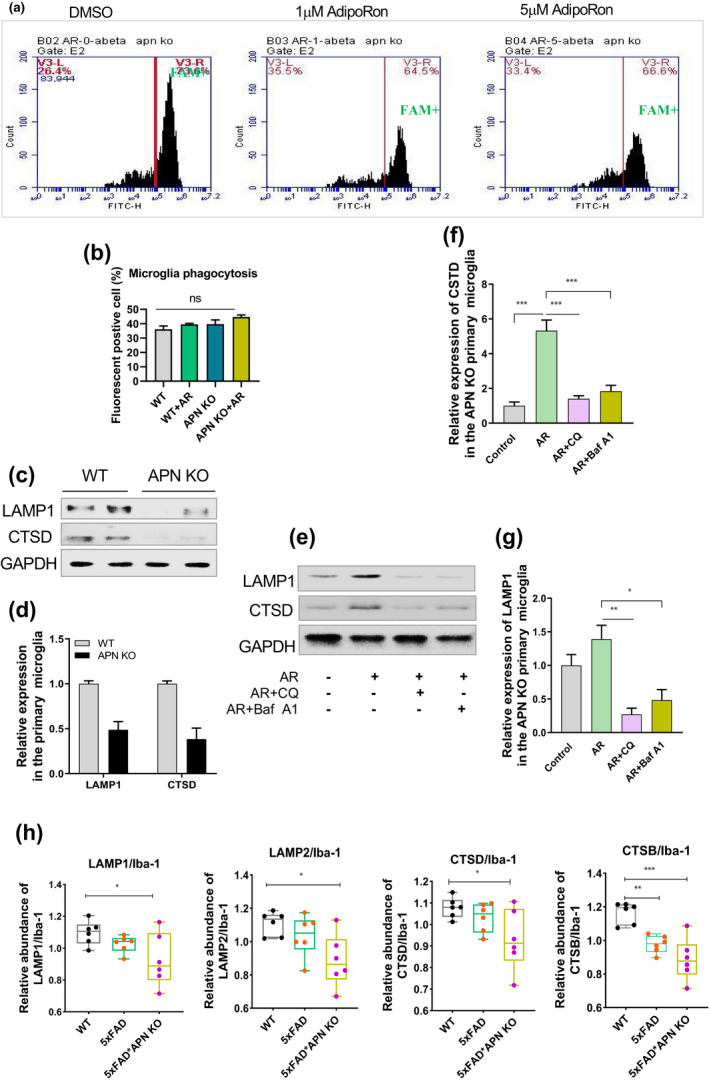
AR promoted Aβ clearance by increasing lysosome activity in microglia. The effect of AR on Aβ accumulation in primary microglia from the brain of APN KO mice. (b) The effect of APN deficiency on the phagocytosis of primary microglia from the WT and APN KO mice. (c‐d) The relative expression of LAMP1 and CTSD in primary microglia from the WT and APN KO mice. (e‐g) The relative expression of LAMP1 and CTSD in primary microglia from the APN KO mice with or without inhibitor treatment. (h) The relative expression of LAMP1, LAMP2, CTSD, and CTSB when the expression abundance of microglia was used as the baseline according to the results from mass spectrometry. Data were expressed as mean ± SEM, ^*^ *p* < 0.05, ^**^ *p* < 0.01, ^***^ *p* < 0.001

To elucidate the underlying mechanisms of microglia activation and Aβ deposits, the effect of AR treatment on Aβ accumulation was measured in primary microglia of APN KO mice. As shown in Figure 6a, AR treatment significantly reduced Aβ accumulation within microglia, indicating that AR might promote Aβ degradation or phagocytosis. Thus, we used fluorescent beads to examine the phagocytic ability of microglia derived from WT or APN‐KO mice (Figure 6b). Furthermore, no apparent differences were observed in microglia's phagocytosis between the two groups (Figure 6b). It indicates that APN deficiency did not affect microglia's phagocytosis.

Interestingly, further results reported that lysosomal markers, including LAMP1 and CTSD (Figure 6c, d), were remarkably reduced in microglia from APN KO mice compared to the WT control, indicating APN deficiency could seriously mitigate the lysosome activity in microglia. These results were further validated with microglia primary culturing of APN KO mice followed by AR treatment; significantly increased lysosomal markers’ expression was detected in the presence of CQ or Baf A1 (Figure 6e, g). Besides, we measured the APN receptors (AdipoR1/2) expression and its downstream signaling molecules, including APPL1 and APPL2 (Figure S7A). Interestingly, AR treatment significantly increased AdipoR1 and APPL1 expression in the hippocampus of the APP/PSI mice. The results were further validated by AR treatment to N2a/APPswe cells (Figure S7B). Furthermore, we overexpressed AdipoR1 and AdipoR2 in the 293T cells and measured the expression of LC3B concurrently (Figure S7E). AdipoR1 overexpressed cells showed an increased level of LC3B, suggesting the involvement of AdipoR1 in the APN‐linked autophagic impairment. As AMPK signaling plays a vital role in autophagy regulation (Kim, Kundu, Viollet, & Guan, 2011), we inhibited the AMPK pharmacologically (via Dorsomorphin) the N2a/APPswe cells (Figure S7D). Surprisingly, AR pro‐autophagy effect was reversed by AMPK antagonism, suggesting AMPK involvement in the APN‐associated autophagy impairment.

Furthermore, we overexpressed AdiopR1 (Figure S8A) in the N2a/APPswe cells and measured the Aβ changes. Cells transfected with AdiopR1 displayed a significant decline in the Aβ_1‐42_ expression (but not Aβ_1‐40_) and Aβ_1‐42_/_1‐40_ ratio (Figure S8B‐D). Notably, we found an increased level of LC3B II in the AdipoR1‐overexpressed cells (Figure S8A). Contrarily AdiopR1 overexpression reduced the Aβ_1‐42_ level in the N2a/APPswe cells. Next, N2a/APPswe cells were treated with an AMPK inhibitor (Dorsomophin or AdipoR1), and Aβ changes were measured. Surprisingly, Dorsomophin treatment reversed the effects of AdipoR1 on Aβ changes (Figure S8E–G).

These findings strongly suggested that APN played an essential role in regulating the lysosomal activity of microglia. Dysregulated APN signaling contributes to AD pathology, whereas AR supplementation could rescue the phenomenon by abating the Aβ in microglia via enhancing lysosomal activity.

## 3 DISCUSSION

The present study demonstrates that APN, an adipokine involved in regulating lipid and glucose metabolism, is significantly reduced in the plasma of dementia patients, including AD and VaD and mice models of AD. Then, 5xFAD*APN KO mice displayed accelerated Aβ deposition accompanied by neuroinflammation and cognitive impairments, which could be rescued by AR treatment. Aβ deposition did not result from APP expression and processing changes but from lysosomal dysregulation and autophagy impairment. These results were further corroborated by AR treatment, which activates AMPK‐mTOR signaling to promote Aβ degradation and eliminates a toxic protein. Finally, the proteomic profile reveals multiple vital biological processes and pathways involved in regulating APN deficiency, confirming APN deficiency, accelerating cognitive impairment, and AD‐like pathologies.

Over the past decade, APN has widely studied in obesity, diabetes, inflammation, atherosclerosis, and cardiovascular disease due to its essential role in regulating glucose and lipid metabolism (Wang & Scherer, 2016). APN had displayed strong anti‐inflammation and oxidative properties in various animal models (Abou‐Samra et al., 2020; Boursereau et al., 2017). However, the detailed involvement of APN in dementia, especially AD, is still insufficient. As some clinical studies indicated, ambiguous results that exist with the APN level showing a decrease, increase, or no significant changes in AD patients (Dukic et al., 2016; Gorska‐Ciebiada et al., 2016; Khemka et al., 2014). Nevertheless, a recent study strongly supported the idea that the low level of APN significantly increased AD risk (Ng et al., 2020). In our study, the plasma APN level was dramatically decreased in dementia patients, exhibiting a negative correlation with the severity of dementia. Also, APN levels demonstrated an age‐related decline in 5xFAD mice. These combined findings further highlighted the etiological contribution of the low APN level in AD.

5xFAD mice characterized by rapid onset and obvious pathological manifestations are widely using as an AD mouse model to evaluate the efficacy or explore the intervention mechanism. Previous studies demonstrated that APN deficiency aggravated cognitive impairment and neuropathology in 9‐month‐old 5xFAD mice (Ng et al., 2020). In the present study, we have focused on earlier stages in AD and showed that APN deficiency accelerated cognitive impairment and AD pathology deterioration in 5xFAD mice. Further, our results supported this observation that APN deficiency may significantly promote AD's early onset, suggesting that APN deficiency may accelerate the pathophysiology of AD. Accumulation of toxic Aβ is one of the factors for the deterioration of cognitive impairment (DeTure & Dickson, 2019), and interfering with the generation and deposition of Aβ may be a strategy for AD treatment. Further mechanistic insight into how APN deficiency aggravated AD pathology and cognitive impairment found that the dysregulation of APN signaling did not alter APP expression and processing, indicating that APN signaling had no pronounced effect on Aβ generation. Thus, aggravated Aβ deposition on APN‐deficient 5xFAD mice suggested that the regulation of APN signaling mainly affected the accumulation and processing of Aβ.

Autophagy is the main conserved pathway for the clearance of abnormal proteins such as toxic Aβ and hyperphosphorylated tau (Li et al., 2017). Several studies showed that autophagy deficits occurred in the early stage of AD and had an essential role in generating and metabolism of Aβ (Uddin et al., 2018). Activation of autophagy was thought to be beneficial in AD treatment (Hamano et al., 2018). Our findings showed that AR treatment could significantly reduce the ratio of Aβ_1‐42_/Aβ_1‐40_ and induced autophagy activation via regulating AMPK‐mTOR signaling, supporting that AR treatment improved AD cognitive impairment at least partially through autophagy activation‐mediated Aβ elimination. Moreover, previous studies have documented that the ALP are involved in eliminating long‐lived protein and dysfunction organelles, and ALP defects result in various abnormalities, including AD (Martini‐Stoica et al., 2016). Besides, many groups have reported that autophagy modulation in nonhuman models of neurological disorder accelerates clearance of diseases causing protein, like toxic Aβ, and improves disease phenotypes (Barmada et al., 2014; Dolan & Johnson, 2010; Finkbeiner, 2020; Jia et al., 2007; Martini‐Stoica et al., 2016; Sarkar et al., 2007; X. Wang et al., 2010). Although the ALP mechanism has been discussed in the AD‐associated pathologies (Orr & Oddo, 2013), up to our knowledge, we did not find reliable evidence of the association between APN deficiency and ALP defects, which inspire us to explore this mechanism. We found that AR treatment significantly recovered the lysosomal functional protein levels in the presence of inhibitors (CQ and BafA1) in microglia from APN KO mice. This finding indicates that APN deficiency might be involved in accelerating ALP defects, followed by Aβ accumulation, which participates in AD progression.

On the contrary, in addition to the increase of Aβ accumulation, there were many other factors of exacerbating cognitive impairment in AD, such as chronic neuroinflammation (Kinney et al., 2018), mitochondrial dysfunction (W. Wang et al., 2020), endoplasmic reticulum stress (Salminen et al., 2009), synaptic dysfunction (Kashyap et al., 2019), and so on. Thus, we further employed a TMT‐labeled proteomic approach (read Supplementary data for a detailed discussion of proteomic study) to find more possible mechanisms that APN deficiency aggravated cognitive impairment. We discovered that APN deficiency strongly affects ALP signaling and higher connectivity among neuron differentiation processes, neurogenesis, proteolysis, cell death, autophagy, and endocytosis detected in the brain tissue of 5xFAD*APN KO compared to 5xFAD WT mice. Whether APN is also involved in the processes mentioned above that eventually contributed to AD etiology still warrant further investigation. However, it strongly suggests the APN and the above‐related proteins may play a crucial role in APN deficiency accelerating cognitive impairment in AD.

## 4 CONCLUSION

To our knowledge, this is the first study that identified the negative role of APN in the progression of AD‐like pathophysiologies via the autophagy‐lysosomal pathway. A defect in this signaling could accelerate Aβ deposition in microglia, which is the hallmark of AD pathologies. Further, AR, a potent APN agonist, could reduce these changes and offer a promising therapeutic avenue for treating dementia, including AD.

## 5 METHODS

### 5.1 Human plasma samples

Blood samples that were collected followed by plasma separation from AD patients (MCI = 39), and the age‐ and sex‐matched nondemented healthy individuals (n = 41) (Table S1, S2) from the First Hospital of Zibo kindly provided by prof. Shujin Wang (Neurology Department, the First Hospital of Zibo, China). Mini‐Mental State Examination (MMSE), Clinical Dementia Rating (CDR) Scale, magnetic resonance imaging (MRI), and NINCDS/ADRDA or NINDS/AIREN criteria (Sarazin et al., 2012; Scheltens & Hijdra, 1998) used to diagnose AD and VaD in the individuals. Notably, the study was approved by the Ethical Committee of the First Hospital of Zibo, and consent from all the subjects was included.

### 5.2 Animal and AR treatment

5xFAD mice (B6. Cg‐Tg(APPSwFlLon, PSEN1*M146L*L286V)6799Vas/Mmjax, kindly provided by prof. Ying Xu from Jinan University, China), APP/PS1 mice (APPswe/PSEN1dE9, kindly provided by prof. Qiang Zhou from Peking University Shenzhen Graduate School, China), and APN KO mice (B6;129‐Adipoq^tm1Chan^/J, purchased from The Jackson Laboratory) were bred and housed at Shenzhen Center for Disease Control and Prevention. APN‐deficient 5xFAD (5xFAD*APN KO) mice generated by crossing 5xFAD and APN KO mice. All mice were maintained on a 12‐h light/dark cycle (lights on at 6:00 am, lights off at 6 pm) with ad libitum access to diet. 3‐month‐old 5xFAD*APN KO mice were daily administrated with 50 mg of AR per kg body weight by oral gavage for 4 months. 6‐month‐old APP/PS1 and WT mice were fed with AR for 2 months in the same way. Experimental animals were randomly divided into control or AR treatment group. The study was approved by the Animal Care and Use Committee of the Experimental Animal Center at Shenzhen Center for Disease Control and Prevention.

### 5.3 Behavior test

All tests were conducted in the behavioral room, beginning at 8:00 am every day as performed in our previous studies (Shen et al., 2020; Zhou et al., 2019). The mice were transferred to the specific room 2 h before the test. Novel object recognition (NOR) and Y‐maze were performed to evaluate short‐term memory, while long‐term memory was assessed by the Morris water maze (MWM) test. A 3‐day window was arranged between different behavioral experiments to avoid interference effects. Detailed behaviors assays are mentioned in the Supplementary data.

### 5.4 Histochemistry

We performed Immunohistochemistry as previously reported (Zhou et al., 2019). Detailed histological analyses are mentioned in the Supplementary data.

### 5.5 Proteomics analysis

#### 5.5.1 Sample preparation and protein labeling

The proteomics study was performed as previously reported (He et al., 2019). Mice hippocampal tissue samples were lysed with 8 M urea containing protease inhibitor cocktail (1X), centrifuged at 4℃, 14000 g for 30 min, followed by collected into a new 1.5 ml centrifuge tube. Each sample's protein concentration was quantified by a Nanodrop 2000 and adjusted to the protein concentration of 1 μg/μl with the lysed buffer. A total of 100 μg of protein from each sample were incubated with 10 mM DTT at 55℃ for 60 min, followed by 25 mM IAA treatment for 60 min at room temperature. Each fully denatured sample was incubated with 4 μg Trypsin/Lys‐C Mix at 37℃ for 3 h in a final concentration of 8 M urea buffer. Then, above reaction was diluted with PBS to reduce urea concentration to 1 M, followed by incubating overnight at 37℃. The digested peptides from each sample were desalted, dried, and finally labeled with TMT 10plex reagents.

#### 5.5.2 Peptide Fractionation

TMT‐labeled samples were fractionated using nanoflow DIONEX UltiMate 3000 RSLCnano System (Thermo Fisher Scientific, USA) coupled with C18 resin (300 Å, 5 μm; Varian, Lexington, MA) and a silica capillary column (75 μm ID, 150 mm length; Upchurch, Oak Harbor, WA). The samples were separated by a gradient from 5% to 90% ACN at a flow rate of 0.30 μl/min. Peptides were separated into 45 fractions, which were consolidated into 15 fractions. The fractions were subsequently dried and re‐dissolved in 0.1% formic acid.

#### 5.5.3 LC‐MS/MS and database searching

LC‐MS/MS analyzed the re‐dissolved peptides with the same LC system equipped with Q Exactive HF‐X Quadrupole‐Orbitrap (Thermo Fisher Scientific, USA) in data‐dependent acquisition (DDA) mode. A single full‐scan mass spectrum obtained the data acquisition in Orbitrap (350–1800 m/z, 70,000 resolution) followed by the top 20 data‐dependent MS/MS scans.

The resultant mass spectrometric data were analyzed using Proteome Discoverer 2.6 (Thermo Fisher Scientific, USA) using a protein database composed of the Mus musculus fasta database downloaded from UniProtKB on Auggust 26 2020, containing 17053 reviewed protein sequences. The enzymes to trypsin were set with two missed cleavage tolerance. The static modifications were then fixed to carbamidomethylation (+57.021464) of methionine and acetylation (+42.010565) of peptides’ N‐termini. Precursor ion mass tolerance was set to 20 ppm. Similarly, the fragment ion mass tolerance was also selected as 20 mmu for all MS/MS spectra obtained. Quantitative precision was expressed with protein ratio variability. Differentially expressed proteins (DEPs) were set at *p* < 0.05.

#### 5.5.4 Bioinformatic analysis

Multiple approaches were employed to analyze proteomic results including SIMCA 14.1 software for principal component analysis (PCA) and partial least squares‐discriminate analysis (PLS‐DA). R‐package of "Mfuzz" and "heatmap" was used for Cluster membership and hierarchical heatmap analysis. Pathway enrichment, followed by RStudio 4.0.0 (for visualization), was conducted via online WEB‐based Gene SeT AnaLysis Toolkit (http://www.webgestalt.org/option.php). The TBtools software was employed to carry out a Venn diagram analysis of three group mice's hippocampal proteome. The protein–protein interaction (PPI) networks were analyzed by using a web‐based tool Omicbean (http://www.omicsbean.cn/).

### 5.6 Cell culture, transfection, and flow cytometry analysis

N2a/APPswe cells (kindly gifted by professor Jian‐zhi Wang, Tongji Medical School, HUST, China) were cultured in 6‐well plates with 40% DMEM and 50% Opti‐MEM supplemented 10% FBS in the presence of 0.2 g/L G418. N2a/WT cells purchased from the Cell Bank of China (CODE: IFO50495, China) were also cultured in 6‐well plates with MEM supplemented by 10% FBS humidified atmosphere of 5% CO_2_ at 37℃. When the density of cells reached 70–80% confluence, N2a/APPswe cells were transferred to a serum‐free medium for continuous culture, followed by incubated for 24 h with AR (1 μM or 5 μM), 3‐MA (5 mM), CQ (40 μM), and AR +3‐MA or AR +CQ association. After incubation, cells were lysed with RIPA buffer containing protease inhibitor cocktail (1X) and then centrifuged at 12000 g for 20 min. The supernatant was quantified via a BCA kit and stored at −80℃ for ELISA and Western blot. For autophagosome analysis, N_2_a/APP_swe_ cells were cultured on the bottom of confocal dishes. When grown to 20–30% confluence, they were transfected with the plasmid expressing mCherry‐GFP‐LC3B using the Lipofectamine 2000 reagent according to the manufacturer's instructions. After 48 h, the transfected cells were treated with 1 µM or 5 µM AR for 24 h. Finally, the expression of mCherry and GFP was visualized using a confocal microscope.

Microglia primary culture was performed according to the previously developed protocol (M. Wang et al., 2020). In brief, brain tissues from postnatal (day 1–3) APN KO mice and WT mice were isolated, cut into tiny pieces, and followed by digestion with trypsin for 15 min. Dissociated tissues mixed with glia were then plated into T‐25 culture flasks containing DMEM/F12 with 10% fetal bovine serum, GlutaMAX (Invitrogen), and 1% penicillin/streptomycin. After culture in a 5% CO_2_/37℃ incubator for 14 days, the flasks were shaken at 220 rpm for 4 h at 37℃ to harvest the primary microglia. After that, the microglia were plated in 6‐well plates at a density of 5 x 10^5^ cells per well for Western blot and flow cytometry analysis. For phagocytosis analysis, primary cultured microglia were treated with AR (1 µM or 5 µM) followed by incubated with FAM‐Aβ_1‐42_ (500 nM) for 2 h. Cells were then washed with PBS and collected for analysis by flow cytometry using BD Accuri C6 Plus and subsequently analyzed by flow cytometry.

### 5.7 Enzyme‐linked immunosorbent assay (ELISA) and Western blot

ELISA kits for human and mouse adiponectin, human Aβ_1‐40,_ and Aβ_1‐42_ were obtained from R&D systems. APN level in the plasma of human dementia patients and experimental animals was quantified. The level of Aβ_1‐40_ and Aβ_1‐42_ in the supernatants from N_2_a/W.T. and N_2_a/APP_swe_ cells with or without different treatments such as AR, 3‐MA, Dorsomorphin (Dor), and AdipoR1 overexpression was measured according to the manufacturer's instructions. The level of inflammatory cytokines from the lysate of N_2_a/APP_swe_ cells with or without AR or Dor treatment was measured from Elascience.

Hippocampal and cell lysate supernatant was obtained and quantified by using the BCA protein assay kit. Protein samples from animals or cells were then added loading buffer and boiled at 100℃ for 8 min to denature the protein. Degenerated protein lysates were isolated by 10% SDS‐PAGE, transferred onto a 0.22 μm PVDF membrane, blocked with 5% nonfat milk, dissolved in 1 x TBST buffer, and incubated overnight with primary antibodies on ice and then with the corresponding secondary antibodies for 1 h at room temperature. Finally, protein chemiluminescence signal was measured by using the ECL kit and quantified using Quantity One 4.6.2 software.

### 5.8 Statistical analysis

The data was presented as the mean ± SEM and analyzed using GraphPad Prism 8.0 statistical software (GraphPad Software, Inc., La Jolla, CA, USA). A two‐tailed unpaired Student's test was applied to compare two groups statistically. Simultaneously, one‐way analysis of variance (ANOVA) and two‐way analysis of variance were employed to determine the statistical significance of differences among groups and follow Dunnett's multiple comparison test. A probability value of *p* < 0.05, *p* < 0.01, and *p* < 0.001 was considered statistically significant.

## CONFLICT OF INTEREST

The authors declare no conflict of interest.

## AUTHOR CONTRIBUTIONS

Kaiwu He and Lulin Nie designed and performed the experiments. Tahir Ali analyzed data and wrote the manuscript. Zizhen Liu, Weifen Li, Kaiqin Zhang, and Jia Xu helped in the experiment. Shujin Wang, Xiao Chen, and Jianjun Liu helped in manuscript, experimental tools and supported the study. Zhi‐Jian Yu, Xifei Yang, and Shupeng Li endorsed the study, corresponding authors, reviewed and approved the manuscript, and held all the responsibilities related to this manuscript. All authors reviewed and approved the manuscript.

## ETHICAL APPROVAL AND CONSENT TO PARTICIPATE

According to the protocols approved by the Animal Care and Use Committee of the Experimental Animal Center at Shenzhen Center for Disease Control and Prevention, all experimental procedures were carried out.

## CONSENT FOR PUBLICATION

All involved parties consented to the publication of this work.

## Supporting information

Fig S1‐S8

Table S1‐S3

Supplementary Material

## Data Availability

All data generated or analyzed during this study are included in this published article [and its supplementary information files].
