# Supplementary material for: Adiponectin alleviated Alzheimer‐like pathologies via autophagy‐lysosomal activation
Source: Aging Cell. 2021 Nov 14;20(12):e13514. doi: 10.1111/acel.13514 (PMC8672778; doi:10.1111/acel.13514)
Supplement: Supplementary file 1 — Fig S1‐S8 [file ACEL-20-e13514-s001.docx]

**Supplementary Figure 1: The effect of APN deficiency on Aβ pathology in 5xFAD mice.**

1. The scheme of building targeted mice (5xFAD*APN KO). (B) Representative images of Aβ plaque with 6E10 antibody in the hippocampus, cortex and amygdala region of 5xFAD and 5xFAD*APN KO mice. (C-F) Quantification of APP, BACE1, ADAM10, and IDE in the hippocampus region. Data were expressed as mean ± SEM, ^*^*p* < 0.05, ^**^*p* < 0.01, ^***^*p* < 0.001, ^****^*p* < 0.0001. Scale bar = 200 μm.

**Supplemental Figure 2. The effect of APN on cognitive impairment in 5xFAD mice.**

(A) The preference of novel object in the new object recognition test. (B) The percentage of time spent in a novel arm in the Y-maze test. (C) The escape latency in the Morris water maze test. (D) The representative swimming trace in the probe trial. E) The percentage of distance traveled in the target quadrant. (F) The percentage of time spent in the target quadrant. (G) Total distance traveled. (H) Average speed. Data were expressed as mean ± SEM, ^*^*p* < 0.05, ^**^*p* < 0.01, ^***^*p* < 0.001.

**Supplemental Figure 3. AR treatment reduced Aβ plaque deposition and neuroinflammation, not changed protein level involved in Aβ processing and production.**

1. Representative images and quantification of Aβ plaque with 6E10 antibody in the hippocampus and cortex of APP/PS1 mice with or without AR treatment. (B) The relative expression of APP, BACE1, ADAM10, and IDE in the hippocampus region of APP/PS1 mice with or without AR treatment. (C) Representative images of GFAP (a marker of astrocyte activation) and quantification in the cortex and hippocampus region of WT, 5xFAD, and 5xFAD*APN KO mice. (D) Representative images of Iba1 (a marker of microglial activation) and quantification in the cortex and hippocampus region of WT, 5xFAD, and 5xFAD*APN KO mice. Data were expressed as mean ± SEM, ^*^*p* < 0.05, ^**^*p* < 0.01. Scale bar = 100 μm.


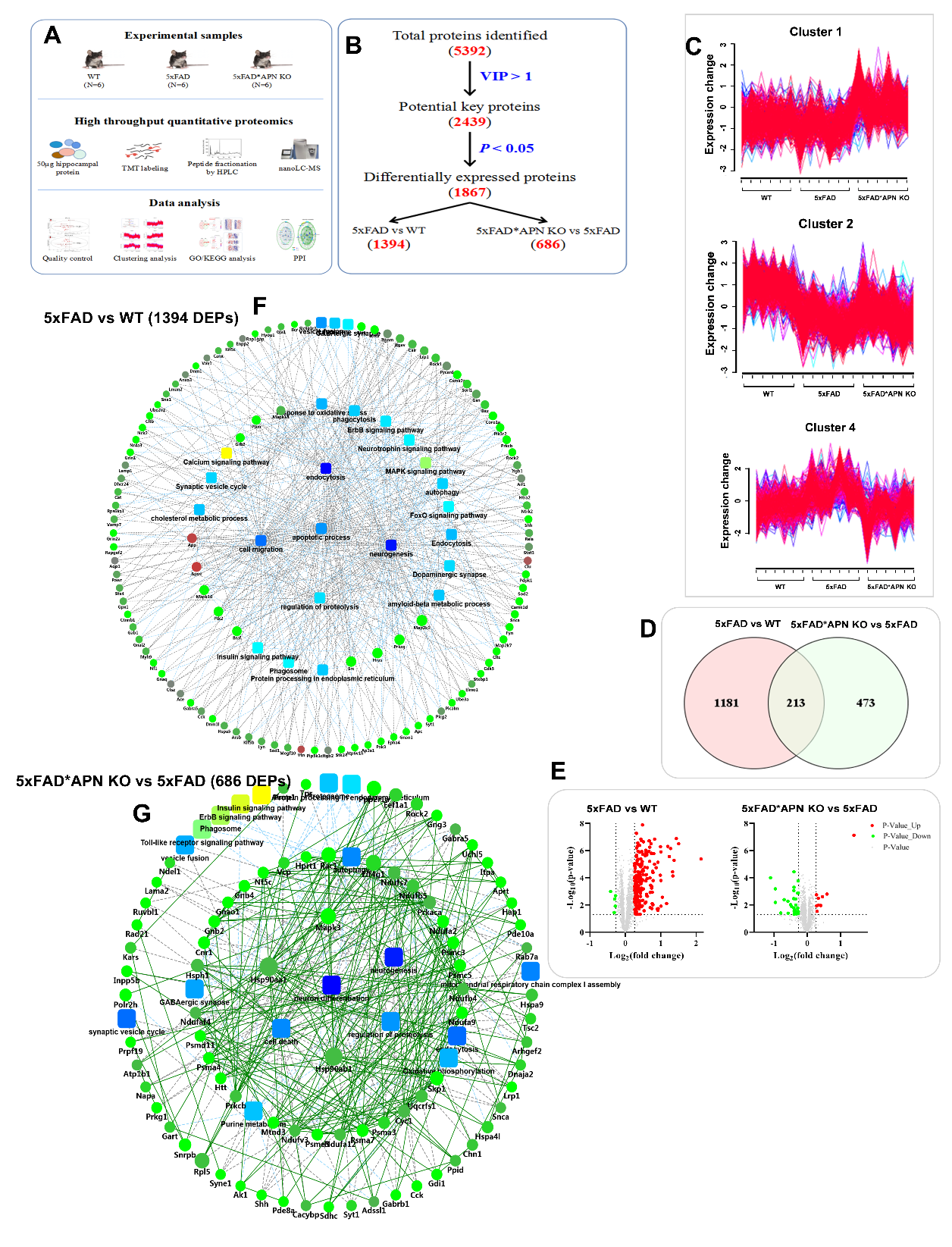


**Supplemental Figure 4. Proteomics analysis.**

1. The flow chart of brain hippocampal proteomics analysis. (B) Overview of the qualitative results. (C) The expression patterns of cluster 1, cluster 2, and cluster 4. (D) The venn diagram analysis of DEPs between 5xFAD vs WT and 5xFAD*APN KO vs 5xFAD. (E) Using a cutoff of fold change >1.2 and p value < 0.05 as standard, volcano plots of 2439 potential key proteins in three group. Plots illustrating the distributions of proteins according to their log_2_(fold change) (x-axis) and −log_10_(p-value) (y-axis). (F-G) The protein-protein network indicated the interactions of DEPs with GO/KEGG term in 5xFAD vs WT and 5xFAD*APN KO vs 5xFAD, respectively. The circle represented the DEPs and the square represented GO/KEGG term. The higher the connectivity, the more important it was.

**Supplemental Figure 5. The effect of AR on autophagy activation in N2aAPPswe cells.**

(A-F) Quantification of Beclin-1, ATG7, ATG5, LC3II/LC3I, pAMPK/AMPK, and pmTOR/mTOR in N2aAPPswe cells with or without AR treatment. (G-H) Quantification of ATG7 and LC3II/LC3I in the presence of 3-MA. (I-J) Quantification of ATG7 and LC3II/LC3I in the presence of CQ. Data were expressed as mean ± SEM, **p* < 0.05, ***p* < 0.01, ****p* < 0.0001.

**Supplemental Figure 6. The effect of APN signaling on Aβ plaque and Aβ metabolism.**

**(A)** The level of APN in plasma of MCI group and normal control. **(B)** The level of Aβ1-40 in the supernatant of N2a/WT and N2a/APPswe cells with or without AdipoRon. **(C)** The level of Aβ plaque with 6E10 antibody in the cortex region of 5xFAD*APN KO mice with or without AdipoRon. **(D)** The effect of APN deficiency on the Aβ metabolism in N2a/APPswe cells. Data were expressed as mean ± SEM, **p < 0.01, ***p < 0.001. Scale bar = 100 μm.

**Supplemental Figure 7.** **The effect of AR treatment on APN signaling molecules, autophagy, and inflammatory factors.**

(A). The effect of AdipoRon on APN signaling molecules including AdipoR1, AdipoR2, APPL1, and APPL2 in the hippocampus of WT and APP/PS1 mice. (B) The effect of AdipoRon on APN signaling molecules including AdipoR1, AdipoR2, APPL1, and APPL2 in N2a/APPswe cells. (C) The level of Aβ1-42 and Aβ1-40 in N2a/APPswe cells. (D) The effect of dorsomorphin treatment on LC3 in N2a/APPswe cells. (E) The effect of overexpressed AdipoR1 and AdipoR2 on LC3 in 293T cells. (F) The level of inflammatory factors in N2a/APPswe cells with or without AdipoRon. Data were expressed as mean ± SEM, *p < 0.05, **p < 0.01.

**Supplemental Figure 8**. **The effect of AdipoR1 transfection and Dorsomophin treatment on Aβ expression**

(A): Representative blots and bar graphs show adiopR1 and LC3B expression in the N2a/APPswe cells. (B, C, and D): Bar Graphs show Aβ-140, Aβ-142, and Aβ-142/140 ratio status, in Dorsomophin (AMPK inhibitor) or AdipoR1 treated N2a/APPswe cells. (E, F, and G): Bar graphs show expression of Aβ-140, Aβ-142, and Aβ-142/140 ratio, in AdipoR1 transfected N2a/APPswe cells. Data were expressed as mean ± SEM, *p < 0.05, **p < 0.01.
