# Supplementary material for: Adiponectin alleviated Alzheimer‐like pathologies via autophagy‐lysosomal activation
Source: Aging Cell. 2021 Nov 14;20(12):e13514. doi: 10.1111/acel.13514 (PMC8672778; doi:10.1111/acel.13514)
Supplement: Supplementary file 2 — Table S1‐S3 [file ACEL-20-e13514-s002.docx]

|  | ***Normal*** | ***MCI*** |
| --- | --- | --- |
| N (male:female) | 41 (17:24) | 39 (17:22) |
| Age (years) | 69.54 ± 0.9203 | 69.67 ± 0.7261 |
| Education (years) | 10.392 ± 4.359 | 11.538 ± 3.924 |
| Mini-cog | 5 | 2.897 ± 0.686 |
| MMSE | 30 | 12.359 ± 0.978 |
| Adiponectin (ng/ml) | 7691.036 ± 31.097 | 5578.811 ± 44.257 |

**Supplementary Table 1: Characteristics of dementia Patients**

MCI: Mild Cognitive impairment, MMSE: Min-Mental State Examination,

**Supplementary Table 2: Characteristics of dementia Patients**

|  | **Nomal** | **AD** | **VaD** |
| --- | --- | --- | --- |
| N(male:female) | 10 (4:6) | 10 (5:4) | 10 (5:4) |
| Age (years) | 75.7 ± 2.343 | 71.22 ± 3.527 | 74.56 ± 1.292 |
| Education (years) | 6.1 ± 1.616 | 7.444 ± 1.324 | 5.778 ± 2.165 |
| ADL | 14.1 ± 0.1 | 26.22 ± 5.098 | 33.78 ± 4.509 |
| MMSE | 26.3 ± 0.9195 | 15.56 ± 3.019 | 17.33 ± 2.392 |
| CDR | 0 ± 0 | 1.611 ± 0.3514 | 1.444 ± 0.3275 |
| Adiponectin (ng/ml) | 7014 ± 805.9 | 4811 ± 606.7 | 3856 ± 685.3 |

MMSE = Min-Mental State Examination. CDR = Clinical Dementia Rating. ADL = Activities of Daily Living Scale.

**Supplementary Table 3: List of antibodies used:**

| Antibody | Source | Cat.No | Application |
| --- | --- | --- | --- |
| 6E10 | BioLegend | 803015 | IHC/IF |
| GFAP | Millipore | MAB360 | IHC/IF/WB |
| Iba1 | Wako | 019-19741 | IHC/IF/WB |
| APP | Abcam | Ab32136 | WB |
| BACE1 | Cell Signaling Technology | 5606S | WB |
| ADAM10 | Abcam | Ab39178 | WB |
| IDE | Abcam | Ab32216 | WB |
| pAMPK | Cell Signaling Technology | 2535S | WB |
| AMPK | Cell Signaling Technology | 2532S | WB |
| pmTOR | Cell Signaling Technology | 2971S | WB |
| mTOR | Cell Signaling Technology | 2972S | WB |
| Beclin-1 | BD Biosciences | 612112 | WB |
| ATG5 | Cell Signaling Technology | 2630S | WB |
| ATG7 | Abcam | Ab133528 | WB |
| LC3B | Cell Signaling Technology | 2775S | WB |
| LAMP1 | Abcam | ab24170 | WB |
| CTSD | Abcam | ab75852 | WB |
| AdipoR1 | Abcam | Ab70362 | WB |
| AdipoR2 | Proteintech | 14361-1-AP | WB |
| APPL1 | Cell Signaling Technology | 3858S | WB |
| APPL2 | Proteintech | 15041-1-AP | WB |
| β-actin | Santa Cruz | sc-47778 | WB |
| α-tublin | Sigma | T-9026 | WB |
| GAPDH | Cell Signaling Technology | 5174S | WB |
