# Supplementary material for: Adiponectin alleviated Alzheimer‐like pathologies via autophagy‐lysosomal activation
Source: Aging Cell. 2021 Nov 14;20(12):e13514. doi: 10.1111/acel.13514 (PMC8672778; doi:10.1111/acel.13514)
Supplement: Supplementary file 3 — Supplementary Material [file ACEL-20-e13514-s003.docx]

**Suplemmatrary data**

**Behaviors assays**

***Novel object recognition (NOR)***

Mice were placed in a white box (50 cm x 50 cm) and acclimated to two identical objects for 10 min. After 60 min, one of the objects replaced with a novel object, and the mice were allowed to explore the objects for 5 min while videotaped. The object exploring time of the mice was defined as time spent exploring with the noseless than 1.5 cm from the objects. Finally, Xeye software was used to measure mice's preference towards novel objects, reflecting the degree of short-term memory.

1. ***maze***

Y-maze test was performed to evaluate the short-term spatial reference memory of the mice. The maze consisted of three identical arms (44 x 10 x 15 cm) positioned 120^o^ apart. In the training session, mice were placed into the apparatus from the start arm and allowed to explore the maze for 15 min with one arm closed off as the novel arm. After 60 mins, the mice were placed back into the maze and allowed mice to explore the maze for 5 min with the blockage removed. The time of mice in the novel arm was recorded to measure the short-term spatial reference memory.

**Morris water maze (MWM)**

MWM test was performed to evaluate the long-term spatial reference memory. The maze was made of a circular tank with a diameter of 1.7 m filled water mixed with no-fat milk (a depth of 0.3 m). The tank was divided into 4 quadrants (I - IV), and a circular white escape platform was placed in the target quadrant 2 cm below the surface of the water maintained at 22 ± 1℃. In the acquisition training, mice were trained for 5 successive days, each day composed of the different sessions with four hidden platform trials. Mouse was given 60 s to find the hidden platform in every trial, and the time to find the hidden platform was defined as the escape latency. If the mouse can not find the platform within 60 s, it will be manually guided to stay on the invisible platform 15 s. After 6 days, the mouse was placed into the first quadrant pool after removing the hidden platform and allowed to explore the pool for 2 min freely. All behavioral parameters of the experimental mice were recorded using the video tracking system.

**Reagents and antibodies**

AdipoRon (AR) was purchased from Tao Su Biochemical Technology Co. Ltd (Shanghai, China). 3-Methyladenine (3-MA), chloroquine phosphate (CQ), bafilomycin A1 (baf A1), and Dorsomorphin (Dor) were purchased from MedChemExpress (MCE, USA). Trypsin/Lys-C Mix was purchased from Promega (Wisconsin, WI, USA). TMT-10plex reagent was purchased from Thermo Fisher Scientific (Waltham, MA, USA). FAM-Aβ_1-42_ and TAMRA-Aβ_1-40_ was purchased from AnaSpec (Fremont, CA, USA). All other chemical reagents mentioned were analytical grade unless otherwise stated. The antibodies used in this study have been listed in Supplementary Table 1.

**Histochemistry**

Mice were deeply anesthetized with 4% chloral hydrate and intracardially perfused with saline. Brains were immediately removed and split into two parts on the mid-sagittal plane. The hippocampus and cortex were separated from the left cerebral hemisphere and stored at -80℃ until use. The right cerebral hemisphere was transferred to fresh 4% paraformaldehyde overnight at 4℃. The fixed right hemibrains were dehydrated by gradient alcohol (75% ethanol, 2 h; 85% ethanol, 1 h; 95% ethanol, 1 h; 95% ethanol, 1 h; 100% ethanol, 0.5 h; 100% ethanol, 0.5 h; xylene, 0.5 h; xylene, 0.5 h; paraffin, 0.5 h; paraffin, 0.5 h; paraffin, 0.5 h), and then quickly embedded into paraffin blocks. 5 µm sections of these brain tissue were collected by using a paraffin microtome. Upon use, the paraffin-embedded sections were carried out deparaffinized and rehydration with xylene and ethanol, followed by antigen retrieval methods to unmask the epitope.

Immunohistochemistry was performed as previously reported (Zhou et al., 2019). Briefly, brain sections were washed (three times with PBS) and incubated for 10 min in Hydrogen Peroxide Block to remove endogenous peroxidase. The sections were then blocked with Protein Block for 60 min at room temperature, followed by primary antibodies including mouse monoclonal anti-6E10, mouse monoclonal anti-GFAP rabbit polyclonal anti-Iba1 overnight at 4℃. The next day, the sections were incubated with Biotinylated Goat Anti-Polyvalent for 60 min, followed by incubation with streptavidin peroxidase for 30 min. Signal was visualized by diaminobenzidine (DAB)-hydrogen peroxide substrate to give a brown color. The images were collected using a light microscope equipped with a digital camera (Olympus BX60, Japan).

For immunofluorescent studies, the sections were washed three times with PBS and blocked with blocking buffer (0.3% Triton X-100 + 3% bovine serum albumin in PBS) for 60 min, followed by primary antibodies including mouse monoclonal anti-GFAP and rabbit polyclonal anti-Iba1 overnight at 4℃. After primary antibodies incubation, the sections were labeled with fluorescent secondary antibodies as follows: Alexa Fluor 488 goat anti-mouse IgG (H+L) and Alexa Fluor 568 goat anti-rabbit IgG (H+L). DAPI (4, 6-diamidino-2-phenylindole) was used to counterstain the nuclei. The images were acquired using a confocal microscope and analyzed using ImageJ Pro Plus software.

**Cell transfection**

To evaluate the effect of AdipoR1 and AdipoR2 overexpression on autopphagy and Aβ accumulation, 293T cells and N_2_a/APP_swe_ cells were transfected with the plasmid overexpressing AdipoR1 and AdipoR2 using the Lipofectamine 2000 reagent, respectively. The transfected cells were lysed with RIPA buffer, then the level of LC3B was measured by western blot, and Aβ accumulation was measured by ELISA.

**Proteomic analysis**

To investigate the possible effects and underlying mechanisms of APN deficiency in AD, proteomic analysis based on TMT-labeled was performed in the hippocampus of WT, 5xFAD, and 5xFAD*APN KO group (**Figure S4A**). We identified a total of 5392 proteins among the three (WT, 5xFAD, and 5xFAD*APN KO) groups. Further, for the proteome difference, we performed the PCA and PLS-DA analysis. The three groups of samples were distinguished in the above two models (**Figure 3A**). We used the PLS-DA model to identify potential vital proteins of possible discriminatory significance based on the magnitude (score) of the variable influence in projection (VIP), where a VIP score > 1.0 indicated a significant contribution to the discriminatory. Proteins showing VIP score > 1.0 with p-value < 0.05 were considered differentially expressed proteins (DEPs). Interestingly, our analysis showed that 2439 potential key proteins and 1867 DEPs, including 1394 DEPs in 5xFAD vs WT group and 686 DEPs in 5xFAD*APN KO vs 5xFAD group (**Figure S4B**). According to different expression patterns, the three groups' 2439 potential key proteins were further divided into 5 clusters (**Figure 3B; Figure S4C**). In cluster 3 the potential key proteins increased significantly in 5xFAD mice than WT mice, further increasing in APN deprived 5xFAD mice. In contrast, cluster 5- related potential key proteins decreased in 5xFAD mice compared to WT and further decreased in APN deficient 5xFAD mice. These results indicated that cluster 3/5- related potential vital proteins might be the molecular basis for APN deficiency exacerbating AD cognitive impairment and pathology in 5xFAD mice.

To further evaluate, we subsequently performed KEGG enrichment analysis and hierarchical heatmap clustering analysis. A total of 631/256 important DEPs were revealed in cluster 3/5 of three groups (**Figure 3C, F**). Interestingly, the KEGG analysis results showed that cluster 3 DEPs were mainly involved in regulating protein processing in the endoplasmic reticulum and lysosome (**Figure 3D**). Then, the DEPs engaged in the two most essential pathways were further analyzed by hierarchical heatmap clustering, as shown in (**Figure 3E**). In cluster 5, the KEGG analysis results showed that these crucial DEPs were mainly involved in regulating proteasome, dopaminergic synapse, and insulin signaling (**Figure 3G**). And the DEPs involved in the three most essential pathways were further visualized by hierarchical heatmap clustering in three groups of mice (**Figure 3H**).

A total of 213 DEPs were screened from the two different sets (**Figure S4D**), suggesting that these DEPs maybe have an essential effect in APN deficiency aggravating AD cognitive impairment. Using a cutoff of fold change >1.2 and p-value < 0.05 as standard, 207 DEPs were found significantly regulated in 5xFAD mice compared with WT mice. In 207 DEPs, 202 DEPs upregulated (marked red dots), whereas 5 DEPs were down-regulated (marked green dots). Furthermore, 9 up and 29 down-regulated in the 38 DEPs, in 5xFAD*APN KO mice compared with 5xFAD mice (**Figure S4E**). To further validate the interactions between the DEPs, we constructed a network of protein interactions using the DEPs of two different sets, respectively. Combined with the biological process link KEGG pathway, the network was divided into three levels according to the connectivity degree. The closer the connection, the more critical it may be.

Compared to higher connectivity among the process of neurogenesis, endocytosis, cell migration, apoptotic process, and proteolysis regulation in 5xFAD mice compared to WT. Moreover, App and Apoe proteins not only had higher connectivity in the network, but also greater fold change in 5xFAD mice compared with WT mice (**Figure S4F**). Similarly, the terms of neuron differentiation, neurogenesis, regulation of proteolysis, cell death, autophagy, and endocytosis were observed to have a higher connectivity in 5xFAD*APN KO mice compared with 5xFAD mice. Notably, some heat shock protein family proteins (Hsp90aa1, Hsp90ab1, Hsph1) and mitochondrial related proteins (Ndufaf4, Ndufb5, Ndufb4, Ndufs7, Ndufv3, Uqcrfs1, Cyc1, etc) had higher connectivity and greater fold change (**Figure S4G**). These data suggested that the above terms and proteins may play a crucial role in APN-deficiency accelerating cognitive impairment in AD.

A detailed discussion of the proteomic study as follows.

**Protein processing in endoplasmic reticulum and lysosome**

In an upregulated expression pattern, protein processing in the endoplasmic reticulum and lysosome involved in DEPs were the two pathways with the most significant influence. Generally, the endoplasmic reticulum (ER) served as the main compartment for the folding, maturation, trafficking, and quality control of newly synthesized proteins (**29129774**). With the continual accumulation of toxic Aβ, a defense mechanism termed "ER stress" was triggered to maintain the normal operation of cells. In turn, severe ER stress could further aggravate the unfolded protein response (UPR) leading to the accumulation and aggregation of misfold proteins, which contributed to the pathogenesis of AD (**27815720**). In this study, compared with WT mice, the ER stress signaling was significantly upregulated in 5xFAD mice, indicating that Aβ deposition initiated cellular ER stress. Although there was no significant change between 5xFAD group and 5xFAD*APN KO group, we could observe an upward trend of ER stress signaling. Therefore, we speculated that ER stress may play an important role in APN-deficiency accelerating cognitive impairment.

AD was characterized by abnormal accumulation of protein aggregates, primarily extracellular plaques composed of the Aβ peptide and intracellular tangles comprised of the tau protein, both of which may indicate a primary defect in protein clearance (**29758300**). As lysosome was the last step to degrade these aggregated proteins, its dysfucnction would be cause impaired lysosomal proteolysis and further accelerate the pathogenesis of AD (**21878991**). Our study found that dysregulation of lysosomal signaling was observed in 5xFAD mice. In particular, some lysosomal regulators such as lysosome-associated membrane glycoprotein 1 (LAMP1) and lysosome-associated membrane glycoprotein (LAMP2) were found to be up-regualted in 5xFAD mice compare WT mice. Consistent with previous studies, the level of LAMP1 and LAMP2 assessed as AD biomarkers was significantly increased in AD individuals when compared with cognitively normal subjects (**24101586**). On the other hand, APN deficiency showed only an increasing trend in levels of lysosomal associated proteins, which may be due to the onset of 5xAD mice at 6 months.

**Proteasome, dopaminergic synapse, and insulin signaling**

In down-regulated experssion pattern, three major pathways including proteasome, dopaminergic synapse, and insulin signaling were identified. Importantly, APN deficiency significantly down-regualted the expression of above signaling pathways associated proteins. In eukaryotes, targeted intracellular protein degradation was largely mediated by the proteasome that composed of a 20S core particle harboring the proteolytic active sites and a 19S regulated substrate binding and targeted protein entry into core particle (**26997268**). The ubiquitin-proteasome system (UPS), the major intracellular protein quality control system, was related to AD pathogenesis (**24512022**). Moreover, the proteasome complex was aslo a major regulator of intracellular protein quality control and an essential proteolytic enzyme for the processing of both Aβ and Tau (**27392670**). In our study, we found that the levels of proteasome subunit alpha type-4 (psma4) and proteasome subunit alpha type-7 (psma7), the two necessary subunits of 20S core particle (**26997268**), and 26S proteasome regulatory subunit 8 (psmc5) (a 19S proteasomal ATPase) (**25962134**) and 26S proteasome regulator subunit 11 (psmd11) ( a 19S proteasomal non-ATPase) (**19616115**) were significantly decreased in APN deficiency 5xFAD mice compared with 5xFAD mice, indicating the decline of proteasome-dependent proteolysis caused by APN deficiency in AD.

Although pathological hallmarks of AD were senile plaques, neurofibrillary tangles, and neuronal degeneration, synaptic loss was an early event in the pathogenesis of AD (**25511446**). We found that levels of a variety of synaptic proteins, particularly dopaminergic synapse-related proteins, were significantly down-regulated in APN deficiency 5xFAD mice. As some studies demonstrated, dopamine was a well-recognized modulator of hippocampal synaptic plasticity and its binding to DAergic receptors in the dorsal hippocampus was a major determinant of memory encoding (**19696353, 25326690, 26904943**). Therefore, we speculated that dopaminergic synapse may be one of the targets of APN regulating cognitive function.

Since APN is an insulin-sensitive adipocytokine, we can predict that APN deficiency may be more largely affect insulin signaling in 5xFAD mice. As expected, the results of proteomics analysis showed that APN deficiency obviously decreased insulin signaling of 5xFAD mice, as indicated by the reduced expression of insulin signaling-related proteins. Notably, decreasing insulin signaling was observed in in post-mortem brain tissue from AD patients, and enhance insulin signaling could improve cognitive function (**29353052**). On the other hand, a previous study demonstrated that AR treatment could enhance insulin sensitive and ameliorate insulin resistance in 5xFAD mice (**32132650**). These findings suggested that insulin signaling pathway played an important role in APN deficiency accelerating cognitive impairment.

**Heat shock protein, proteasomal proetins and mitochondrial related proteins**

Importantly, we found the the three categories of DEPs had higher connectivity with other DEPs, indicating that changes in their expression may play a crucial role in cognitive function involved in APN deficiency. For example, heat shock proteins (Hsp90aa1 and Hsp90ab1), proteasomal proteins (Psma3, Psma4, Psma7, Psmc3, Psmc5, Psme3, and Psmd11), and mitochondrial related proteins (Ndufa2, Ndufa9, Ndufa12, Ndufaf4, Ndufb4, Ndufb5, Ndufs7, Ndufv3, Mtnd3, Uqcrfs1, and Cyc1). Firstly, HSP90, an ATP-dependent chaperone, was viewed to be involved in the regulation of AD pathogenesis, and it had multiple properties including facilitating metastable protein maturation, stabilizing aggregation-prone proteins, and quality control of misfolded proteins (**29203712**). Numerous studies demonstrated that overexpressed HSP90 could reduce toxic Aβ aggregation (**20166963, 30200516**). And exocytosis of HSP90 further stimulate microglial activation to pushing Aβ clearance in nervous system (**11919167**). Our data showed that APN deficiency significantly reduced the level of HSP90, indicating the involvement of HSP90 in APN deficiency exacerbating Aβ pathology. Secondly, proteasomal proteins were necessary for proteasome-dependent proteolysis as discussed earlier. Our study showed that the levels of multiple proteasomal proteins were significantly reduced in APN deficiency 5xFAD mice compared with 5xFAD mice, indicating that APN deficiency seriously affected the structure and function of proteasome in 5xFAD mice. Thirdly, mitochondrial dysfunction had been identified as an early event in AD pathogenesis and was reflected by reduced metabolism, disruption of Ca^2+^ homeostasis, and increased levels of reactive oxygen species, lipid peroxidation, and apoptosis (**29551631**). ATP production was often regarded as an important indicator of mitochondrial function, which required the involvement of multiple proteins associated with the electron transport chain including complexes (I-IV) (**26537901**). The results of peotein-protein interactions showed that mitochondrial related proteins, especially complex I-related proteins, were in the position of high degree of connectivity in whole network, and their level of expression was almost decreased in APN deficiency 5xFAD mice, suggesting that APN deficiency mainly affected mitochondrial complex I in 5xFAD mice.
